# Cardiovascular disease risk under low-fat, Mediterranean, and American Heart Association diets: a target trial emulation in United States adults

**DOI:** 10.1016/j.ajcnut.2026.101404

**Published:** 2026-06-20

**Authors:** Yi Li, Dalia Stern, Miguel A Martínez-González, Shuyuan Yang, Jorge E Chavarro, JoAnn E Manson, Eric B Rimm, Robert W Platt, Yu-Han Chiu

**Affiliations:** 1 Department of Epidemiology, Biostatistics, and Occupational Health, School of Population and Global Health, Faculty of Medicine, McGill University, Quebec, Canada; 2 SECIHTI-Population Health Research Center, National Institute of Public Health, Cuernavaca, Mexico; 3 Department of Preventive Medicine and Public Health, University of Navarra, Pamplona, Spain; 4 Instituto de Salud Carlos III, Centro de Investigacion Biomédica en Red Fisiopatología de la Obesidad y Nutrición (CIBERObn), Madrid, Spain; 5 Department of Nutrition, Harvard T.H. Chan School of Public Health, Boston, MA, United States; 6 Department of Epidemiology, Brown University School of Public Health, Providence, RI, United States; 7 Center for Gerontology and Health Care Research, Brown University School of Public Health, Providence, RI, United States; 8 Department of Epidemiology, Harvard T.H. Chan School of Public Health, Boston, MA, United States; 9 Channing Division of Network Medicine, Department of Medicine, Brigham and Women’s Hospital and Harvard Medical School, Boston, MA, United States; 10 Division of Preventive Medicine, Department of Medicine, Brigham and Women’s Hospital and Harvard Medical School, Boston, MA, United States

**Keywords:** Mediterranean diet, low-fat diet, AHA-2020 dietary goals, target trial emulation, parametric g-formula, cardiovascular disease risk

## Abstract

**Background:**

The Mediterranean diet reduces cardiovascular disease (CVD) risk in European populations, but its long-term effectiveness in United States adults, particularly relative to the American Heart Association (AHA)-2020 dietary goals, remains uncertain.

**Objectives:**

We compared 20-y CVD risk in United States adults under sustained adherence to a low-fat diet, a Mediterranean diet, and the food-based AHA-2020 dietary goals.

**Methods:**

We emulated a target trial using data from the Nurses’ Health Study and Health Professionals Follow-up Study among 12,197 United States adults aged 55 to 80 y with diabetes or ≥3 major CVD risk factor. Diet was assessed every 4 y via validated food frequency questionnaires. The primary outcome was CVD, defined as nonfatal myocardial infarction, coronary revascularization, stroke, and CVD death, confirmed by medical records. We applied the parametric g-formula to estimate 20-y CVD risk under each dietary strategy.

**Results:**

Over 20 y, 3469 CVD cases occurred (1451 men and 2018 women). Pooled absolute risks of CVD were 35.9% (95% CI: 33.1%, 38.5%) under the low-fat diet, 28.2% (95% CI: 25.8%, 31.4%) under the Mediterranean diet, and 31.2% (95% CI: 29.1%, 33.4%) under the AHA-2020 dietary goals. Compared with the low-fat diet, estimated risk ratios were 0.79 (95% CI: 0.70, 0.91) for the Mediterranean diet and 0.87 (95% CI: 0.79, 0.96) for the AHA-2020 dietary goals. In a general population not selected for age, diabetes, or CVD risk factors, the corresponding risk ratios were 0.90 (95% CI: 0.84, 0.95) and 0.94 (95% CI: 0.90, 0.99), respectively.

**Conclusions:**

Sustained adherence to the Mediterranean diet and the AHA-2020 dietary goals is estimated to have a lower 20-y risk of CVD than a low-fat diet in United States adults.

## Introduction

The Mediterranean diet is widely recognized as one of the healthiest diets for cardiovascular disease (CVD) prevention [1]. Observational studies have consistently shown associations between Mediterranean dietary patterns and a lower risk of CVD [[2], [3], [4], [5], [6], [7]], although the definitions of these dietary patterns vary across studies. In the landmark PREDIMED (Prevención con Dieta Mediterránea) trial, which enrolled Spanish individuals aged 55 to 80 y at high cardiovascular disease risk, participants assigned to a Mediterranean diet supplemented with extra-virgin olive oil or mixed nuts had a lower incidence of major CVD than those randomly assigned to a low-fat diet [8]. Whether these findings extend to United States populations, and how they compare with other healthy dietary patterns such as those recommended by the American Heart Association (AHA), remains unclear [9]. Given recent stagnation in declines of CVD incidence and mortality among younger adults in the United States [10], it is also critical to evaluate the effectiveness of Mediterranean diets in a broader population.

Because conducting another randomized trial in the United States may be infeasible, we emulated a target trial modeled after PREDIMED, with adaptations for United States diets [11,12]. We compared 3 strategies: the low-fat diet, the Mediterranean diet, and the AHA-2020 dietary goals. Using data from 2 large observational cohort studies, we estimated CVD risk under each strategy in high-risk individuals (i.e., older adults with type 2 diabetes or multiple CVD risk factors) and extended the analysis to the general population, not selected by age, diabetes status, or baseline CVD risk factors. We hypothesized that sustained adherence to the Mediterranean diet and AHA-2020 dietary goals would reduce 20-y risk of CVD in United States adults.

## Methods

Target trial emulation is a framework that explicitly aligns the design and analysis of an observational study with a hypothetical randomized trial [11,12]. In this study, we aimed to compare 3 strategies: a low-fat diet and a Mediterranean diet, both informed by the PREDIMED trial [8] and the AHA-2020 dietary goals.^9^ The eligibility criteria were also designed to broadly align with those of the PREDIMED trial. To define these interventions under study, we first described the PREDIMED trial and the AHA-2020 dietary goals, which informed the specification of the dietary strategies in the target trial and its subsequent emulation.

### The PREDIMED trial: the Mediterranean diet compared with the low-fat diet

The PREDIMED trial randomly assigned 7447 older adults in Spain at high cardiovascular disease risk but free of CVD at enrollment between 2003 and 2009 to 1 of 3 diets: a Mediterranean diet with extra-virgin olive oil, a Mediterranean diet with mixed nuts, or a low-fat diet [8], Participants were instructed to adhere to their assigned diet throughout follow-up. The Mediterranean diet encouraged olive oil, nuts, fatty fish, legumes, and sofrito, whereas the low-fat diet encouraged low-fat dairy and discouraged fats and oils for spreading, dressing salads, or cooking; fried snacks and nuts; visible fat in meats and soups; commercial sweets or pastries; and fatty fish. Adherence to the diets was assessed annually by a questionnaire [13]. The primary endpoint was a composite of myocardial infarction, stroke, and death from cardiovascular causes. Over a median follow-up of 4.8 y, CVD risks were 3.8% in the Mediterranean diet with extra-virgin olive oil group, 3.4% in the Mediterranean diet with nuts group, and 4.4% in the low-fat diet group. The hazard ratio for the combined Mediterranean diets compared with the low-fat diet was 0.70 (95% CI: 0.55, 0.89) in the intention-to-treat analysis and 0.42 (95% CI: 0.24, 0.63) in the per-protocol analysis [7].

### The AHA-2020 dietary goals

The AHA-2020 dietary goals included 5 primary metrics: fruits and vegetables; fish; whole grains; sodium; and sugar-sweetened beverages, and 3 secondary metrics: nuts, legumes, and seeds; processed meat; and saturated fat [9]. More recent AHA dietary guidance removed nutrient-based targets and added recommendations favoring plant oils over animal fat, low-fat dairy over full-fat dairy, and limiting alcohol [14], while remaining broadly consistent with the 2020 dietary goals. Because the newer guideline did not specify quantitative intake targets, our emulation focused on 6 food-based components of the AHA-2020 dietary goals (Supplemental Table 1) [8].

### Specification of the protocol of (hypothetical) target trial

We specified a hypothetical target trial comparing 3 strategies: a low-fat diet, a Mediterranean diet, and the food-based AHA-2020 dietary goals (Table 1, third column), described below.

**TABLE 1:** Summary of the protocols for the PREDIMED trial, the target trial, and its observational emulation

	The PREDIMED trial	The target trial	Target trial emulation using NHS and HPFS
Eligibility	Inclusion criteria: Men (55–80 y of age) or women (60–80 y of age) who met ≥1 of the following 2 criteria: a) Type 2 diabetes mellitus b) ≥3 of the following major risk factors: • Current smoking • Hypertension • Elevated LDL cholesterol • Low HDL cholesterol • Overweight or obesity • A family history of premature coronary artery disease (before 55 y in fathers or before 65 y in mothers) Exclusion criteria 1 A history of cardiovascular disease (CVD), including coronary artery disease, stroke (ischemic, hemorrhagic, or transient ischemic attack) or peripheral artery disease with intermittent claudication 2 Severe medical condition that may impair the ability for participation in a nutrition intervention study 3 Illegal drug use, chronic alcoholism or total daily alcohol intake of >80 g/d 4 BMI> 40 kg/m^2^ 5 Impossibility to follow a Mediterranean-type diet, for religious reasons or due to disorders of chewing or swallowing 6 Any other medical condition thought to limit survival to <1 y 7 Immunodeficiency or HIV-positive status 8 A low predicted likelihood to change dietary habits 9 History of food allergy with any of the components of olive oil or nuts 10 Participation in any drug trial 11 Institutionalized patients for chronic care 12 Illiteracy 13 Patients with an acute infection or inflammation were allowed to participate in the study 3 mo after the resolution of their condition	Inclusion criteria: same as the PREDIMED trial with the following modifications: • Elevated LDL and low HDL cholesterol are taken as hypercholesterolemia, • A family history of premature coronary artery disease is taken as a family history of coronary artery disease before 60 y in fathers or mothers Exclusion criteria: 1 Same as the PREDIMED trial 2 Severe medical conditions, defined as those who were diagnosed with Parkinson disease, amyotrophic lateral sclerosis, multiple sclerosis, Crohn disease, and ulcerative colitis 3 Total daily alcohol intake of >80 g/d 4 Same as the PREDIMED trial 5 Vegans, defined as individuals reporting zero intake of red meat, chicken, and seafood at baseline.	Same as the target trial, with the following additional exclusions: • Participants who did not complete questions on diet or covariates • Participants who reported implausible energy intake (<800 or >4200 kcal/d in men; <500 or >3500 kcal/d in women) at prebaseline and baseline questionnaires
Dietary strategies	Each individual would be assigned to 1 of the 3 following strategies, which they would have to follow until the end of the trial: 1) The low-fat diet (with small nonfood gifts) 2) The Mediterranean diet supplemented with extra-virgin olive oil 3) The Mediterranean diet supplemented with nuts • Participants assigned to a dietary strategy are expected to maintain their dietary intake within the range prespecified by the corresponding intervention. • No calorie restriction. • Adherence is assessed annually using a dietary questionnaire. It assumed that each dietary questionnaire accurately reflects the average diet over the past year.	Each individual would be assigned to 1 of the 3 following strategies (Supplemental Table 1) adapted from the PREDIMED trial and the American Heart Association (AHA) 2020 dietary goals, which would be followed for 20 y: 1) The low-fat diet: based on PREDIMED trial recommendations, with modifications excluding sofrito and adjusting olive oil intake to a threshold of ≥1 tbsp/d. 2) The Mediterranean diet: based on PREDIMED trial recommendations, with modifications excluding sofrito and adjusting olive oil intake to a threshold of ≤1 tbsp/d. 3) The AHA-2020 dietary goals • Participants assigned to a dietary strategy are expected to maintain their dietary intake within the range prespecified by the corresponding intervention. • No calorie restriction. • Adherence is assessed annually using a food frequency questionnaire.	Same as the target trial, except • Adherence was assessed every 4 y using a food frequency questionnaire.
Assignment	Participants are randomly assigned to a dietary strategy and are aware of it	Same as PREDIMED trial	We attempted to emulate randomization by adjusting for prebaseline or baseline covariates: baseline age at enrollment, a family history of myocardial infarction before 60 y, smoking status, physical activity, BMI, aspirin use, and prebaseline values of dietary variables and total energy intake.
Outcome	Primary endpoint: a composite of first occurrence of nonfatal myocardial infarction (including coronary revascularization), stroke, or death from CVD. Secondary endpoint: coronary heart disease (fatal coronary heart disease and nonfatal myocardial infarction), stroke (fatal and nonfatal), CVD mortality, and all-cause mortality	Same as PREDIMED trial (including coronary revascularization)	Same as the target trial
Follow-up	Starts at baseline visit (between 2003 and 2009) and ends at death, occurrence of a primary endpoint event, loss to follow-up, or administrative end of follow-up (December 2010), whichever occurs first	Same as PREDIMED trial, except that baseline is set in 1994, and administrative end of follow-up is January 2014	Same as the target trial • Baseline was defined as the date of return of the 1994 questionnaire. • Loss to follow-up was defined as questionnaire nonresponse or incomplete responses to dietary questions.
Causal contrast of interest and key identifying conditions^1^	Intention-to-treatment effect: • Consistency • Positivity • Conditional exchangeability for loss to follow-up. Per-protocol effect • Consistency • Positivity • Conditional exchangeability for loss to follow-up. • Conditional exchangeability for adherence • No measurement error^2^	Intention-to-treatment effect: • Consistency • Positivity • Conditional exchangeability for loss to follow-up. Per-protocol effect • Consistency • Positivity • Conditional exchangeability for loss to follow-up. • Conditional exchangeability for adherence • No measurement error^2^	The effect had all individuals adhered to each dietary strategy throughout the follow-up period. • Consistency • Positivity • Conditional exchangeability for loss to follow-up. • Conditional exchangeability for adherence • No measurement error for adherence. We assumed that each 4-y dietary questionnaire accurately reflects the average diet during the previous 4-y period. • To implement threshold intervention, we assume that at each time interval k, an individual’s natural value of diet corresponds to the diet they would have consumed at that time had the intervention been discontinued right before k.
Statistical analysis	Intention-to-treatment analysis: Kaplan–Meier cumulative incidence curves according to assignment group. Per-protocol effect analysis: adherence was defined as a score of 10 or higher on the 14-item questionnaire for the Mediterranean group or a score or 6 or higher on the 9-item questionnaire for the low-fat group. Apply inverse probability weighting or g-formula to adjust for postrandomization prognostic factors	Intention-to-treatment analysis: Kaplan–Meier cumulative incidence curves according to assignment group. Per-protocol analysis: Apply g-formula to compare the 20-y cumulative incidence of CVD under joint interventions on assignment and adherence, with adjustment for prebaseline and postbaseline prognostic factors associated with adherence and loss to follow-up.	Apply g-formula to compare the 20-y cumulative incidence of CVD under each strategy, with adjustment for prebaseline and postbaseline prognostic factors associated with adherence and loss to follow-up.

1 Identifying conditions are assumptions required for causal identification, even in an infinite sample; modeling assumptions (e.g., no model misspecification) pertain to estimation and are not included in this table.

2 Adherence to dietary interventions in trials is often assessed using self-reported measures and, therefore, relies on the assumption of sufficiently accurate measurement of dietary intake.

### Eligibility criteria

Similar to the PREDIMED trial, eligible participants would be men aged 55 to 80 y and women aged 60 to 80 y who have either type 2 diabetes or ≥3 major CVD risk factors (smoking, hypertension, hypercholesterolemia, overweight or obesity, or a family history of coronary artery disease before age 60 y in fathers or mothers). Exclusion criteria would include a history of CVD, stroke, peripheral artery disease with intermittent claudication, severe medical conditions (e.g., Parkinson disease, amyotrophic lateral sclerosis, multiple sclerosis, Crohn disease, and ulcerative colitis), alcohol intake of >80 g/d, BMI (in kg/m^2^) of ≥40, or baseline vegetarian diet.

### Dietary strategies

Participants would be randomly assigned to 1 of the 3 dietary strategies. The low-fat and Mediterranean diets would follow the recommendations in PREDIMED [8], with modifications: *1*) sofrito removed from both the low-fat and the Mediterranean diets because it is not commonly consumed in the United States; *2*) given the low olive oil intake in the United States, the olive oil recommendation for the Mediterranean diet would be reduced from ≥4 tbsp/d to ≥1 tbsp/d; and *3*) no recommendation for olive oil as main culinary fat. The AHA strategy followed food-based 2020 goals. The specific recommended diets for each strategy are summarized in Supplemental Table 1. No calorie restriction was instructed. Participants would be expected to maintain their dietary intake within the range prespecified by the corresponding intervention [15,16].

### Intervention assignment

Participants would be aware of the assigned strategy.

### Outcomes

The primary endpoint would be major CVD events, defined as a composite of nonfatal myocardial infarction (MI), fatal coronary heart disease (CHD), coronary revascularization, total stroke (fatal and nonfatal), and death from CVD. Secondary endpoints would be total stroke, CHD (a composite of nonfatal myocardial infarction and fatal CHD events), CVD mortality, and all-cause mortality.

### Follow-up

Follow-up comprised from baseline in 1994 until the first occurrence of the event of interest, death, loss to follow-up, or administrative end of follow-up in June 2014, whichever happened first.

### Causal contrasts

The causal contrasts of interest would be the intention-to-treat effect and the effect if all individuals had adhered to the dietary strategy throughout the follow-up [17,18].

### Target trial emulation

The above trial is unlikely to be conducted, and thus, we emulated it using data from 2 prospective cohort studies: the Nurses’ Health Study (NHS) and the Health Professionals Follow-up Study (HPFS).

### Data sources: NHS and HPFS

The NHS (established 1976; *n* = 121,701) and HPFS (established 1986; *n* = 51,529) are ongoing prospective cohort studies of United States female registered nurses and male health professionals [[19], [20], [21], [22]]. In both cohorts, information on lifestyle factors, a medical history, and incident disease was collected via biennial self-administered questionnaires, with a response rate of >90% for each follow-up questionnaire. Diet was assessed using a validated food frequency questionnaire (FFQ) [[21], [22], [23], [24], [25], [26]], first administered to NHS participants in 1984 and 1986 and to HPFS participants in 1986, and subsequently every 4 y. The FFQ initially included an open question about the type of cooking oil. A question about olive oil used as salad dressing was added to FFQ in 1990. Since 1994, 3 items on olive oils (olive oil use in food or bread, salad dressing, and cooking) have been consistently included.

Incident CVD was identified in biennial questionnaires and confirmed by physician-investigators blinded to the participant questionnaire data using medical record review [27,28]. Nonfatal myocardial infarction and stroke were confirmed according to the WHO criteria [29] and the National Survey of Stroke criteria [30], respectively. Coronary bypass and coronary angioplasty was self-reported [27]. Deaths were identified through a search on the National Death Index, supplemented by reports from next of kin and the United States postal system (>98% ascertainment) [31]. Cause of death was defined according to the International Classification of Diseases, eighth revision and ninth revision. Fatal CVD events were confirmed or refuted by reviewing hospital records, death certificates, or autopsy records. The study protocol was approved by the institutional review boards of Brigham and Women’s Hospital and Harvard T.H. Chan School of Public Health; return of questionnaires implied informed consent.

### Emulation of the target trial protocol

We defined baseline as 1994 (the first year when all 3 questions on olive oil consumption were included in the FFQ) and prebaseline as 1990 to adjust for prior diet. We identified participants in the abovementioned cohorts who met all eligibility criteria specified in the target trial protocol. We also excluded participants with incomplete baseline diet or covariate questions or implausible energy intake (<800 or >4200 kcal/d in men; <500 or >3500 kcal/d in women) at prebaseline or baseline to prevent extreme values from affecting the analyses. We used the FFQ to measure dietary adherence over follow-up. Food group definitions are provided in Supplemental Table 2. Eligible individuals were followed up from the return of the baseline questionnaire in 1994 until the event of interest, death, nonreturn of a questionnaire, or 20 y after baseline, whichever occurred first. Because FFQs in our cohorts were collected every 4 y, we assumed each FFQ accurately reflects the average diet during the previous 4 y.

### Statistical analysis

For observational emulation, our causal estimand of interest was the 20-y CVD risk that would have been observed if all individuals in the eligible population had adhered to each dietary strategy throughout the follow-up period [32]. We estimated this quantity using the parametric g-formula because adherence to dietary strategies is time varying and may be influenced by postbaseline factors [33,34]. The parametric g-formula is a generalized form of standardization in which the outcome risk under each dietary strategy is standardized to the joint distribution of baseline and time-varying confounders [33,34]. Briefly, we began by fitting the parametric models to estimate time-varying conditional densities of covariates, diet, and outcomes as a function of past covariate and dietary histories, using models pooled over time. We then used Monte Carlo simulation and the estimated model parameters to generate counterfactual outcome distributions under each dietary strategy. Finally, we calculated the cumulative incidence of the outcome under each strategy by averaging over all generated subject-specific histories.

Baseline covariates included age at enrollment, sex, a family history of myocardial infarction and diabetes, smoking status, BMI, physical activity, diabetes, hypertension, hypercholesterolemia, aspirin use at baseline, and prebaseline (i.e., 1 questionnaire before baseline) values of fruits and vegetables, fish, chicken, red meat, whole grains, wine, sugar-sweetened beverages, high-fat dairy, legumes, nuts, sweets, spread fats, olive oil, and total energy intake. Details of time-varying covariates, their functional forms, and model specification in the g-formula are provided in Supplemental Table 3. These covariates were selected a priori based on their established roles [[35], [36], [37], [38], [39], [40], [41], [42], [43], [44], [45], [46]] as cardiovascular disease risk factors and their potential to confound the relationship between diet and cardiovascular outcomes, including demographic characteristics, lifestyle factors, clinical conditions and disease, and prior dietary intake (see directed acyclic graph, Supplemental Figure 1). Time-varying covariates were included to account for time-varying confounding, such as newly diagnosed conditions that might trigger changes in diet while independently influencing CVD risk. Prebaseline dietary variables were included to capture long-term dietary patterns prior to baseline, which may influence both cardiovascular outcomes and are associated with dietary intake throughout follow-up. Aspirin use and menopausal hormone therapy were included as time-varying covariates because their initiation or discontinuation may reflect changes in cardiovascular disease risk profile, health care contact, and broader preventive health behavior, which could influence both subsequent dietary choices and CVD risk. To address potential confounding by prior total energy consumption without adjusting a potential intermediate variable (i.e., concurrent total energy intake) , we included lagged total energy intake as a time-varying covariate. This approach accounts for prior caloric needs while allowing concurrent total energy intake to remain on the causal path [47,48].

When a time-varying covariate was not assessed in all 2-y periods (e.g., dietary variables), we carried forward the value from the most recent measurement and a product term between the most recent measurement and the time since that measurement. This approach leverages the repeated-measures structure of the data, assuming that prior values are strong predictors of subsequent values over short intervals, while maintaining computational feasibility within the parametric g-formula. To minimize potential bias from prolonged missingness, participants were censored after 2 consecutive missing measurements.

We also conducted the analyses separately for NHS and HPFS. Percentile-based 95% confidence intervals (CIs) were obtained using 500 bootstrap samples.

To assess the sensitivity of the estimates to residual confounding, we *1*) additionally adjusted for asymptomatic screening (≥4 of: routine physical examinations, rectal examination, eye examination, fasting blood sugar, mammography, or sigmoidoscopy/coloscopy for screening); *2*) redefined the primary CVD outcome excluding coronary revascularization as health-conscious participants may undergo this procedure electively; and *3*) used 3 types of skin cancers (basal cell carcinoma, squamous cell carcinoma, and melanoma) as negative outcome controls. Although diet may have some influence on skin cancer risk, these outcomes are primarily driven by noncardiovascular risk factors (e.g., UV exposure) and may reflect nonspecific healthy-user behaviors and screening patterns.

To assess gross model misspecification, we *1*) compared the natural course estimates (i.e., the diet that preserves the dietary distribution of the study population) by inverse probability weighting compared with the parametric g-formula [49]; *2*) adjusted for covariates from previous questionnaires without concurrently measures; *3*) applied different functional forms for covariates; *4*) reordered time-varying covariates reported within the same questionnaire; and *5*) additionally include interaction terms between time-varying dietary variables.

All analyses were conducted using SAS 9.4 and R software. The GFORMULA 4.0 macro is available at http://www.hsph.harvard.edu/causal/software.

### A target trial with broader eligibility

To understand whether the dietary interventions are as effective in a general population, we emulated a second target trial by relaxing the eligibility criteria to include men and women not selected on the basis of advanced age, type 2 diabetes, or CVD risk factors at baseline.

### Patient and public involvement

Participants and the public are not invited to comment on the study design, develop patient–relevant outcomes, interpret the results, or write or edit the manuscript. However, participants were invited to provide constructive feedback on biennial questionnaires across follow-up, which has been incorporated when appropriate.

## Results

### Baseline characteristics

A total of 12,197 individuals (8497 women and 3700 men) at high cardiovascular disease risk were included (Supplemental Figure 2). Baseline characteristics are shown in Table 2. The average proportion of participants meeting each dietary recommendation is reported in Supplemental Table 4.

**TABLE 2:** Baseline characteristics of the high-risk and the general populations

Characteristics	High-risk population^2^		General population^3^	
	NHS^1^ (*n* = 8497)	HPFS^1^ (*n* = 3700)	NHS^1^ (*n* = 62,671)	HPFS^1^ (*n* = 26,709)
	Mean (SD) or %			
Age (y)	65.1 (3.2)	65.7 (6.4)	60.2 (7.1)	60.8 (9.4)
BMI (kg/m^2^)	28.2 (4.2)	27.5 (3.2)	25.9 (4.5)	25.8 (3.2)
Total physical activity (h/wk)	3.1 (3.3)	6.5 (6.8)	2.9 (3.1)	7.2 (6.9)
Caucasian/white (%)	97.6	91.2	97.9	92.2
Family history of myocardial infarction (at age < 60 y) (%)	42.0	27.6	25.1	12.0
Current smoker (%)	16.2	11.6	12.5	5.7
Hypertension (%)	82.7	77.3	34.9	29.8
High cholesterol (%)	85.6	79.0	49.3	40.0
Type 2 diabetes (%)	10.6	11.8	3.0	1.9
Menopausal hormone user (%)	29.3	NA	39.5	NA

1 Abbreviations: HPFS, Health Professionals Follow-up Study; NHS, Nurses’ Health Study.

2 The high-risk population: men aged 55–80 y and women aged 60–80 y with diabetes or ≥3 major cardiovascular disease risk factors at baseline.

3 The general population: not selected based on age, diabetes status, or cardiovascular disease risk factors at baseline.

### Primary endpoints

**FIGURE 1:**
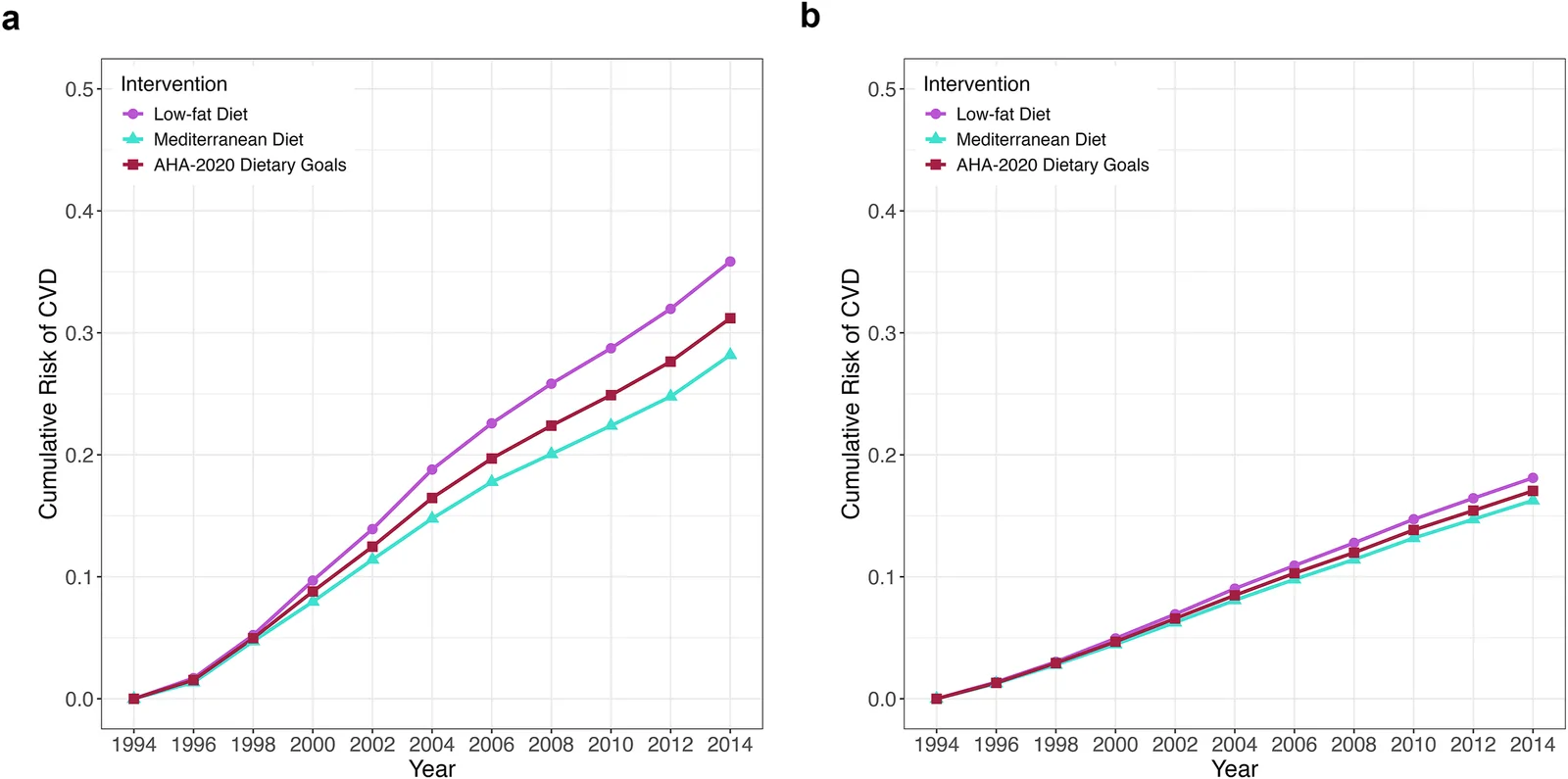
CVD incidence under 3 diets in high-risk and general populations. (A) The high-risk population: men aged 55 to 80 y and women aged 60 to 80 y with diabetes or ≥3 major cardiovascular disease risk factors at baseline. (B) The general population: not selected based on age, diabetes status, or cardiovascular disease risk factors at baseline.

The pooled 20-y estimated absolute risks of CVD were 35.9% (95% CI: 33.1%, 38.5%) under the low-fat diet, 28.2% (95% CI: 25.8%, 31.4%) under the Mediterranean diet, 31.2% (95% CI: 29.1%, 33.4%) under the AHA-2020 dietary goals (Figure 1A; Table 3). Compared with the low-fat diet, the risk ratio was 0.79 (95% CI: 0.70, 0.91) for the Mediterranean diet and 0.87 (95% CI: 0.79, 0.96) for the AHA-2020 dietary goals. The corresponding absolute risk differences were −7.7% (95% CI: −11.2%, −3.2%) and −4.7% (95% CI: −8.0%, −1.4%), respectively. When comparing the Mediterranean diet with AHA-2020 dietary goals, the risk ratio was 0.90 (95% CI: 0.83, 1.00), with an absolute risk difference of -3.0% (95% CI: −5.5%, −0.0%).

**TABLE 3:** Estimated^2^ cumulative risk of endpoints under each dietary strategy in high-risk patients

	Low-fat diet	Mediterranean diet	AHA-2020 dietary goals^1^
**Primary endpoint: major CVD**^1^**events**^3^			
Nurses’ Health Study			
20-y absolute risk (%)	28.1 (24.9, 32.5)	26.0 (21.9, 30.9)	24.8 (21.8, 29.1)
Risk ratio	1.0 (ref)	0.92 (0.76, 1.09)	0.88 (0.77, 1.04)
Risk difference (%)	0.0 (ref)	−2.2 (−6.9, 2.2)	−3.3 (−7.0, 1.0)
Health Professionals Follow-up Study			
20-y absolute risk (%)	46.3 (41.2, 51.4)	35.8 (30.6, 40.8)	41.1 (37.5, 45.2)
Risk ratio	1.0 (ref)	0.77 (0.64, 0.93)	0.89 (0.78, 1.01)
Risk difference (%)	0.0 (ref)	−10.6 (−17.7, −2.8)	−5.2 (−11.0, 0.5)
Combined 2 cohorts			
20-y absolute risk (%)	35.9 (33.1, 38.5)	28.2 (25.8, 31.4)	31.2 (29.1, 33.4)
Risk ratio	1.0 (ref)	0.79 (0.70, 0.91)	0.87 (0.79, 0.96)
Risk difference (%)	0.0 (ref)	−7.7 (−11.2, −3.2)	−4.7 (−8.0, −1.4)
**Secondary end points: stroke (fatal + nonfatal)**			
Nurses’ Health Study			
20-y absolute risk (%)	8.8 (6.9, 11.9)	8.6 (6.2, 12.0)	9.5 (7.8, 12.8)
Risk ratio	1.0 (ref)	0.97 (0.66, 1.41)	1.08 (0.81, 1.47)
Risk difference (%)	0.0 (ref)	−0.3 (−3.6, 3.3)	0.7 (−2.1, 3.6)
Health Professionals Follow-up Study			
20-y absolute risk (%)	6.9 (4.5, 10.1)	6.6 (4.0, 10.7)	7.8 (6.1, 10.7)
Risk ratio	1.0 (ref)	0.95 (0.49, 1.80)	1.14 (0.78, 1.77)
Risk difference (%)	0.0 (ref)	−0.3 (−4.4, 4.3)	1.0 (−2.1, 4.1)
Combined 2 cohorts			
20-y absolute risk (%)	8.2 (6.7, 10.0)	7.4 (5.7, 9.4)	8.3 (7.1,10.0)
Risk ratio	1.0 (ref)	0.91 (0.66, 1.23)	1.02 (0.80, 1.30)
Risk difference (%)	0.0 (ref)	−0.7 (−3.2, 1.7)	0.1 (−2.0, 2.2)
**Coronary heart disease**^4^			
Nurses’ Health Study			
20-y absolute risk (%)	11.3 (9.2, 14.1)	9.4 (6.9, 12.3)	8.9 (7.5, 11.1)
Risk ratio	1.0 (ref)	0.84 (0.58, 1.15)	0.79 (0.60, 1.06)
Risk difference (%)	0.0 (ref)	−1.9 (−5.3, 1.5)	−2.3 (−5.2, 0.5)
Health Professionals Follow-up Study			
20-y absolute risk (%)	19.7 (16.1, 24.4)	13.4 (10.2, 17.4)	19.2 (16.7, 23.2)
Risk ratio	1.0 (ref)	0.68 (0.48, 0.97)	0.98 (0.77, 1.28)
Risk difference (%)	0.0 (ref)	−6.2 (−12.0, −0.5)	−0.5 (−5.6, 4.7)
Combined 2 cohorts			
20-y absolute risk (%)	13.5 (11.4, 15.5)	9.7 (8.1, 12.0)	11.9 (10.4, 13.8)
Risk ratio	1.0 (ref)	0.72 (0.58, 0.93)	0.88 (0.74, 1.07)
Risk difference (%)	0.0 (ref)	−3.8 (−6.1, −0.8)	−1.6 (−3.8, 0.9)
**CVD mortality**			
Nurses’ Health Study			
20-y absolute risk (%)	16.7 (13.9, 20.2)	10.1 (7.2, 13.4)	11.5 (9.5, 14.4)
Risk ratio	1.0 (ref)	0.61 (0.43, 0.82)	0.69 (0.55, 0.88)
Risk difference (%)	0.0 (ref)	−6.6 (−10.8, −2.8)	−5.2 (−8.3, −1.8)
Health Professionals Follow-up Study			
20-y absolute risk (%)	23.7 (19.2, 28.2)	16.1 (12.2, 19.5)	22.9 (19.5, 26.1)
Risk ratio	1.0 (ref)	0.68 (0.49, 0.88)	0.97 (0.80, 1.12)
Risk difference (%)	0.0 (ref)	−7.6 (−13.5, −2.4)	−0.8 (−5.4, 4.5)
Combined 2 cohorts			
20-y absolute risk (%)	18.3 (16.0, 20.6)	11.0 (9.1, 13.4)	14.3 (12.6, 16.6)
Risk ratio	1.0 (ref)	0.60 (0.49, 0.75)	0.78 (0.67, 0.93)
Risk difference (%)	0.0 (ref)	−7.3 (−10.1, −4.3)	−4.0 (−6.6, −1.2)
**All-cause mortality**			
Nurses’ Health Study			
20-y absolute risk (%)	43.9 (40.3, 47.1)	34.0 (30.5, 38.5)	34.7 (32.2, 38.4)
Risk ratio	1.0 (ref)	0.78 (0.68, 0.89)	0.79 (0.73, 0.89)
Risk difference (%)	0.0 (ref)	−9.9 (−14.5, −4.6)	−9.2 (−12.6, −4.7)
Health Professionals Follow-up Study			
20-y absolute risk (%)	55.4 (50.4, 60.2)	45.0 (40.5, 49.7)	49.9 (47.0, 54.1)
Risk ratio	1.0 (ref)	0.81 (0.71, 0.91)	0.90 (0.83, 1.00)
Risk difference (%)	0.0 (ref)	−10.4 (−16.9, −4.6)	−5.5 (−10.0, 0.2)
Combined 2 cohorts			
20-y absolute risk (%)	47.0 (44.7, 49.7)	36.1 (32.9, 39.1)	38.9 (36.7, 41.2)
Risk ratio	1.0 (ref)	0.77 (0.69, 0.83)	0.83 (0.77, 0.88)
Risk difference (%)	0.0 (ref)	−11.0 (−14.9, −7.5)	−8.2 (−11.1, −5.3)

Values in parenthesis are 95% CI.

1 Abbreviations: AHA, American Heart Association; CVD, cardiovascular disease.

2 Estimates based on the parametric g-formula with baseline and prebaseline covariates: baseline age, BMI, smoking status, physical activities, parental history of myocardial infarction, aspirin use, prebaseline intake of fruits, vegetables, fish, whole grains, chicken, red meat, fish, legumes, nuts, high-fat dairy, whole grains, sugar-sweetened beverages, wine, olive oil, sweet, spread fat, and calories; and time-varying covariates: BMI, number of cigarettes smoked, physical activity, aspirin use, menopausal hormone therapy (Nurses’ Health Study only), intake of fruits, vegetables, fish, whole grains, chicken, red meat, fish, legumes, nuts, high-fat dairy, whole grains, sugar-sweetened beverages, wine, olive oil, sweet, spread fat, removing visible fat from meat, and calories, and incidence of high blood pressure, high cholesterol, diabetes, cancer, and time since report of each disease. In the analysis combining 2 cohorts, we additionally adjusted for sex.

3 Major cardiovascular event was defined as a composite of myocardial infarction, fatal coronary heart disease, stroke, death from cardiovascular cause, and coronary revascularization (e.g., coronary artery bypass grafting and percutaneous coronary intervention).

4 Coronary heart disease is defined as a composite of nonfatal myocardial infarction and fatal coronary artery disease.

### Secondary endpoints

Compared with the low-fat diet, the pooled risk ratio for the Mediterranean diet was 0.72 (95% CI: 0.58, 0.93) for coronary artery disease, 0.60 (95% CI: 0.49, 0.75) for CVD-related mortality, 0.77 (95% CI: 0.69, 0.83) for all-cause mortality, and 0.91 (95% CI: 0.66, 1.23) for stroke. The AHA-2020 dietary goals showed similar but weaker associations: compared with the low-fat diet, the pooled risk ratios for the AHA-2020 dietary goals were 0.88 (95% CI: 0.74, 1.07) for coronary artery disease, 0.78 (95% CI: 0.67, 0.93) for CVD-related mortality, 0.83 (95% CI: 0.77, 0.88) for all-cause mortality, and 1.02 (95% CI: 0.80, 1.30) for stroke (Table 3).

### Subgroup analyses

When comparing the Mediterranean diet with the low-fat diet, results were similar across subgroups defined by baseline age, BMI, and physical activity, with cutoffs based on the median. When comparing AHA-2020 dietary goals with the low-fat diet, the magnitude of the risk ratio was greater among participants aged ≥65 y (risk ratio: 0.82; 95% CI: 0.72, 0.93) than those <65 y (risk ratio: 0.96; 95% CI: 0.82, 1.11) (Supplemental Figure 3).

### Sensitivity analyses

Estimates were similar across most sensitivity analyses (Supplemental Table 5). When the primary outcome was redefined as a composite of nonfatal myocardial infarction (MI), fatal coronary heart disease (CHD), and total stroke, absolute risks were ∼10% lower and risk ratios slightly attenuated: the risk ratios were 0.80 (95% CI: 0.68, 0.97) for the Mediterranean diet compared with the low-fat diet and 0.92 (95% CI: 0.81, 1.07) for the AHA-2020 dietary goals compared with the low-fat diet.

### Results for the general population

In the trial emulation with broader eligibility (Supplemental Figure 4), the risk ratio compared with the low-fat diet was 0.90 (95% CI: 0.84, 0.95) for the Mediterranean diet and 0.94 (95% CI: 0.90, 0.99) for AHA-2020 dietary goals (Table 4). Corresponding absolute risk differences were −1.9% (95% CI: −3.0%, −0.8%) for the Mediterranean diet and −1.1% (95% CI: −2.0%, −0.1%) for the AHA-2020 dietary goals.

**TABLE 4:** Estimated^2^ cumulative risk of endpoints under each dietary strategy in general population

Major CVD^1^ events	Low-fat diet	Mediterranean diet	AHA-2020 dietary goals^1^
Nurses’ Health Study			
20-y absolute risk (%)	14.2 (13.3, 15.3)	12.6 (12.0, 13.8)	13.6 (12.8, 14.6)
Risk ratio	1.0 (ref)	0.89 (0.81, 0.97)	0.95 (0.89, 1.04)
Risk difference (%)	0.0 (ref)	−1.6 (−2.8, −0.4)	−0.7 (−1.7, 0.5)
Health Professionals Follow-up Study			
20-y absolute risk (%)	27.7 (25.9, 29.7)	24.6 (22.9, 26.5)	25.5 (24.3, 26.7)
Risk ratio	1.0 (ref)	0.89 (0.82, 0.97)	0.92 (0.85, 0.99)
Risk difference (%)	0.0 (ref)	−3.1 (−5.2, −0.7)	−2.2 (−4.2, −0.2)
Combined 2 cohorts			
20-y absolute risk (%)	18.1 (17.3, 19.0)	16.3 (15.4, 17.2)	17.0 (16.4, 17.8)
Risk ratio	1.0 (ref)	0.90 (0.84, 0.95)	0.94 (0.90, 0.99)
Risk difference (%)	0.0 (ref)	−1.9 (−3.0, −0.8)	−1.1 (−2.0, −0.1)

Values in parenthesis are 95% CI.

Values in parenthesis are 95% CI.

1 Abbreviations: AHA, American Heart Association; CVD, cardiovascular disease.

2 Estimates based on the parametric g-formula with baseline and prebaseline covariates: baseline age, BMI, smoking status, physical activities, parental history of myocardial infarction, aspirin use, prebaseline intake of fruits, vegetables, fish, whole grains, chicken, red meat, fish, legumes, nuts, high-fat dairy, whole grains, sugar-sweetened beverages, wine, olive oil, sweet, spread fat, and calories; and time-varying covariates: BMI, number of cigarettes smoked, physical activity, aspirin use, menopausal hormone therapy (Nurses’ Health Study only), intake of fruits, vegetables, fish, whole grains, chicken, red meat, fish, legumes, nuts, high-fat dairy, whole grains, sugar-sweetened beverages, wine, olive oil, sweet, spread fat, removing visible fat from meat, and calories, and incidence of high blood pressure, high cholesterol, diabetes, cancer, and time since report of each disease. In the analysis combining 2 cohorts, we additionally adjusted for sex.

## Discussion

### Principal findings

In 2 large United States cohorts, we estimated that among older adults at high CVD risk, the 20-y absolute risk of CVD would be 7.7 (95% CI: 3.2, 11.2) percentage points lower under the Mediterranean diet and 4.7 (95% CI: 1.4, 8.0) percentage points lower under the AHA-2020 dietary goals, than a low-fat diet. Benefits were observed in coronary artery disease, CVD-related mortality, and all-cause mortality, but not for stroke. This may reflect differences in underlying pathophysiology and outcome definitions, as stroke is a heterogeneous endpoint and our definition included both ischemic and hemorrhagic subtypes, which may attenuate overall associations [50,51]. Although some heterogeneity in effect estimates was observed between men and women, the direction of associations was generally consistent, and differences in magnitude may reflect variation in baseline cardiovascular disease risk profiles and dietary patterns [52,53]. Results in the general population were consistent, although risk differences were smaller.

### Comparison of findings with the PREDIMED trial

The PREDIMED trial in Spain reported a 2.1% risk reduction in 4.8-y CVD risk with a Mediterranean diet compared with a low-fat diet. If follow-up in our study had been stopped at 4 y, the estimated risk difference would have been −0.5% (95% CI: −1.2%, 0.3%), markedly smaller than in PREDIMED. Some factors may explain the differences. First, because olive oil intake was much lower and sofrito intake was unavailable in our population, we excluded sofrito and reduced the olive oil recommendations from 4 to 1 tbsp/d, likely attenuating benefits of the Mediterranean diet given the importance of extra-virgin olive oil in PREDIMED [54,55]. Second, unlike PREDIMED, which provided free extra-virgin olive oil, nuts, or small nonfood gifts, along with dietary advice on other food items, our dietary strategies made no distinction on intervention delivery methods. Third, baseline diet differed: NHS and HPFS participants reported lower baseline intakes of olive oil, fruit, wine, nuts, fish/seafood, and poultry, but higher intakes of red meat, butter/margarine, and sweets [56]. Fourth, baseline risk factors also differed, with higher prevalence of hypercholesterolemia and a family history of myocardial infarction, but lower prevalence of type 2 diabetes in our cohorts. Finally, PREDIMED may have overestimated the effects due to early termination following an interim analysis [57].

### AHA recommendations compared with PREDIMED

Because the PREDIMED trial tested a traditional Spanish Mediterranean diet rooted in customary culinary practices, we also evaluated the food-based AHA-2020 dietary goals, which align with Mediterranean recommendations but may be more applicable to the United States population. Our estimates suggest that both strategies reduced CVD risk, although the Mediterranean diet showed greater risk differences. Key differences include the following: *1*) PREDIMED emphasized the use of olive oil and moderate wine consumption for current drinkers, whereas the AHA-2020 dietary goals did not; *2*) PREDIMED recommended higher intake of nuts and legumes (6 compared with 4 servings/wk), and seafood (3 compared with 2 servings/wk); and *3*) PREDIMED’s red meat allowances were less restrictive (<7 compared with <2 servings/wk of red processed meat). These differences may partially explain the greater benefit observed for the Mediterranean diet. More recent AHA-2020 dietary also highlights plant oils, low-fat dairy, and limiting alcohol, but without specific intake targets [14].

### Comparisons with observational studies and meta-analyses

Prior analyses in the NHS and HPFS cohorts found higher adherence to a Mediterranean diet score, when compared with low adherence, was associated with ∼20% to 30% reduction in CVD events [6,58]; 15% to 25% reduction in all-cause mortality [59]; and 40% reduction in CVD-mortality [6]. Meta-analysis of 24 studies also showed that higher adherence to the Mediterranean diet was associated with a reduced incidence of CVD, myocardial infarction, stroke, cardiovascular mortality, and all-cause mortality [60]. Our findings are consistent but not directly comparable, as prior work contrasted high compared with low adherence to a Mediterranean diet score rather than comparing 2 distinct dietary strategies.

### Strengths and limitations

Strengths of this study include explicit specification of dietary intervention with a clear start point, duration, and follow-up, consistent with pragmatic randomized trials. Unlike dietary index score methods that compare high compared with low adherence to a single dietary pattern, our approach estimates absolute risks under distinct dietary strategies [61]. Additional strengths include long follow-up, repeated validated assessment of diet and lifestyle and rich covariate information.

Our study also has several limitations. We could not emulate the same amount of olive oil levels (or quantify the proportion of olive oil corresponding to the extra-virgin variety) provided in the PREDIMED trial, although our modified recommendations are informative for developing recommendations that are feasible for the United States population. Residual or unmeasured confounding is possible, despite extensive adjustment of time-fixed and time-varying confounders and sensitivity analyses. In negative control outcome analyses, estimates were close to the null for basal cell carcinoma and imprecise for melanoma; however, the estimate for squamous cell carcinoma was above the null for the AHA-2020 dietary goals compared with the low-fat diet. This finding suggests that residual confounding cannot be excluded, and the magnitude of the estimated protective associations should be interpreted with caution. In addition, because few participants simultaneously met all dietary criteria, estimation of sustained adherence to these strategies relies on parametric modeling assumptions (including limited interactions between dietary variables) and may involve some degree of model extrapolation. Nevertheless, our estimates remained consistent when incorporating selected interactions and alternative functional forms, and natural course estimates were similar across 2 different modeling approaches (Supplemental Figure 5). Although FFQs in these cohorts have been validated against biomarkers and diet records [21,22,[24], [25], [26]] measurement error remains a potential limitation. FFQs are likely to be subjected to both random and systematic measurement error, which may be more pronounced than in other dietary instruments [62,63]. For continuous dietary exposures, the direction and magnitude of bias due to measurement error are difficult to predict, particularly in the context of time-varying, multicomponent interventions [64]. Our analyses therefore relied on the standard working assumption that measurement error is limited; violations of this assumption may influence the magnitude of the estimated associations and their 95% CIs. Finally, participants were predominantly white health professionals with potentially higher health consciousness, which may limit generalizability to more diverse populations.

## Conclusions

We estimate that adhering to a Mediterranean diet and food-based AHA-2020 dietary goals would reduce the 20-y risk of CVD compared with the low-fat diet in United States adults at high cardiovascular disease risk and in a more general population not selected based on age, diabetes, or CVD risk factors.

## Author contributions

The authors’ responsibilities were as follows – YL, Y-HC: conceived and designed the study, developed the analytic plan, conducted data processing and statistical analyses, and drafted the manuscript; DS, MAM-G, SY, JEC, JEM, EBR, RWP: contributed methodological guidance; and all authors: reviewed, revised, and approved the final manuscript.

## Declaration of generative AI and AI-assisted technologies in the writing process

During the preparation of this work, the authors used ChatGPT (GPT-5.5 Thinking; OpenAI) to assist with grammar checking and word-count reduction. The authors reviewed and edited the content as needed and take full responsibility for the content of the publication.

## Data availability

The analytic code is available at https://github.com/einsley1993/MedDiet_CVD. The procedures to obtain and access data from the Nurses’ Health Studies, Nurses’ Health Studies II, and Health Professionals Follow-up Study are described at https://www.nurseshealthstudy.org/researchers (contact e-mail: nhsaccess@channing.harvard.edu) and https://sites.sph.harvard.edu/hpfs/for-collaborators/.

## Funding

The research reported in this publication was supported by the National Institute of General Medical Sciences (NIGMS) of the National Institutes of Health (R35GM154888). The cohorts were supported by research grants UM1 CA186107, U01 CA167552, U01 CA176726, U01 HL145386, R01 HL034594, R01 HL35464, R01 HL088521, and P01 CA87969 from the National Institutes of Health. Y-HC is supported by NIGMS R35GM154888. The content is solely the authors' responsibility and does not necessarily represent the official views of the National Institutes of Health.

## Conflict of interest

EBR is on the scientific advisory board for Wilder supermarket store, Alma Food, and for Goode Health and receives grant funding from the USDA and the National Institutes of Health. None of the funding sources are directly related to the contents of this paper. All other authors report no conflicts of interest.
