# Supplementary material for: Cardiovascular disease risk under low-fat, Mediterranean, and American Heart Association diets: a target trial emulation in United States adults
Source: Am J Clin Nutr. 2026 Jun 20;124(2):101404. doi: 10.1016/j.ajcnut.2026.101404 (PMC13494157; doi:10.1016/j.ajcnut.2026.101404)
Supplement: Supplementary file 1 [file mmc1.docx]

**Cardiovascular risk under low-fat, Mediterranean, and AHA diets: a target trial emulation in US adults**

Yi Li^1^, MS, Dalia Stern^2^, PhD, Miguel A. Martínez-González^3,4,5^, MD, PhD, Shuyuan Yang^9,10^, PhD, Jorge E. Chavarro^5,6,7^, MD, ScD, JoAnn E. Manson^6,7,8^, MD, DrPH, Eric B. Rimm^5,6,7^, ScD, Robert W. Platt^1^, PhD, Yu-Han Chiu^9,10^, MD, ScD

^1^ Department of Epidemiology, Biostatistics, and Occupational Health, School of Population and Global Health, Faculty of Medicine, McGill University, Quebec, H3A 0G4, Canada

^2^ SECIHTI-Population Health Research Center, National Institute of Public Health, Cuernavaca, Mexico

^3^ University of Navarra, Department of Preventive Medicine and Public Health, Irunlarrea 1, 31008 Pamplona, Spain

^4^ Instituto de Salud Carlos III, Centro de Investigacion Biomédica en Red Fisiopatología

de la Obesidad y Nutrición (CIBERObn), Madrid, Spain

^5^ Department of Nutrition, Harvard T.H. Chan School of Public Health, Boston, MA 02115, USA

^6^ Department of Epidemiology, Harvard T.H. Chan School of Public Health, Boston, MA 02115, USA

^7^ Channing Division of Network Medicine, Department of Medicine, Brigham and Women’s Hospital and Harvard Medical School, Boston, MA 02115, USA

^8^ Division of Preventive Medicine, Department of Medicine, Brigham and Women’s Hospital and Harvard Medical School, Boston, MA 02115, USA

^9^ Department of Epidemiology, Brown University School of Public Health, Providence, RI, 02903, USA

^10^ Center for Gerontology and Health Care Research, Brown University School of Public Health, Providence, RI, 02903, USA

**Supplemental Table 1. Summary of three dietary strategies: low-fat, Mediterranean, and AHA dietary goals.**

|  | **The low-fat diet** ^a^ | | **The Mediterranean diet** ^b^ | | **The food-based AHA-2020 dietary goals** ^c^ | |
| --- | --- | --- | --- | --- | --- | --- |
| **Fruits and vegetables** | Fresh fruits | ≥ 3 servings/day | Fresh fruits | ≥ 3 servings/day | Fruits and vegetables ^d^ | ≥ 4.5 servings/day |
|  |  |  |  |  |  |  |
|  | Vegetables | ≥ 2 servings/day | Vegetables | ≥ 2 servings/day |  |  |
| **Grains** |  |  |  |  | Whole grains | ≥ 3 servings/week |
| **Dairy** | Exclusively low-fat dairy | Yes |  |  |  |  |
| **Protein foods** | Seafood | ≤ 1 serving/week | Seafood | ≥ 3 servings/week | Seafood | ≥ 2 servings/week |
|  | Remove fat from meat | Yes |  |  |  |  |
|  | Red and processed meat | ≤ 1 serving/week | Red and processed meat | < 1 serving/day | Red and processed meat | ≤ 2 servings/week |
|  |  |  | White meats instead of red meat ^e^ | Yes |  |  |
|  | Nuts | ≤ 1 serving/week | Nuts | ≥ 3 servings/week | Legumes and nuts | ≥ 4 servings/week |
|  |  |  | Legumes | ≥ 3 servings/week |  |  |
| **Olive oils** | Olive oils | ≤ 1 tbsp/day | Olive oils | ≥ 1 tbsp/day |  |  |
| **Beverages** |  |  | Soft drinks | < 1 drink/day | Soft drinks | ≤ 3 servings/week |
|  |  |  | Wines, optionally, only for individuals who drank alcohol at baseline | ≥ 1 glasses/day, ≤ 3 glasses/day |  |  |
| **Others** | Commercial bakery goods, sweets, and pastries | ≤ 1 serving/week | Commercial bakery goods, sweets, and pastries | < 3 servings/week |  |  |
|  | Spread fats | ≤ 1 serving/week | Spread fats | < 1 serving/day |  |  |

a. The dietary strategy in the control diet group of the PREDIMED trial. The original dietary recommendations for the low-fat diet also include sofrito ≤ 2 servings/wk.

b. The dietary strategy in the experimental group of the PREDIMED Trial. The original dietary recommendations for the Mediterranean diet group include sofrito ≥ 2 servings/wk, olive oil ≥ 4 tbsp/wk, and using olive oil as main culinary fat.  Because olive oil intake is the US population is low, we modified the olive recommendation to ≥ 1 tbsp/day.

c. The original AHA 2020 Dietary Goals also included sodium < 1500 mg/day and saturated fat < 7% of total energy. We focused on food-based AHA-2020 dietary goals because sodium and saturated fat are less well-defined targets and more prone to measurement errors.

d. If individuals would eat less than 4.5 servings of fruits and vegetables per day under no intervention, then individuals would be instructed to eat 2.5 servings of vegetables and 2 servings of fruits per day.

e. If individuals would eat 1 or more servings of red and processed meat per day under no intervention, then individuals would be instructed to eat 1 serving of red and processed meat and additional servings of red and processed meat would be replaced by white meat.

**Supplemental Table 2.** Definition of food groups used for the low-fat diet, the Mediterranean diet, and the AHA 2020 dietary goals

| **Food groups** | **Food components included in the three dietary recommendations** | **Example foods in the food frequency questionnaires** |
| --- | --- | --- |
| **Fruits and**  **vegetables** | **Fruits** | Raisins or grapes, prunes, bananas, cantaloupe, watermelon, fresh apples or pears, oranges, grapefruit, strawberries, blueberries, peaches or apricots or plums, orange juice, apple juice, prune juice, other juice. |
|  | **Vegetables** | Tomatoes, tomato juice, tomato sauce, broccoli, cabbage, cauliflower, brussels sprouts, carrots, mixed vegetables, yellow or winter squash, eggplant or zucchini, yams or sweet potatoes, spinach cooked, spinach raw, kale or mustard or chard greens, iceberg or head lettuce, romaine or leaf lettuce, celery, mushrooms, beets, alfalfa sprouts, garlic, corn |
| **Grains** | **Whole grains** | Whole grain breakfast cereal, other cooked breakfast cereal, cooked oatmeal, dark bread, rye or pumpernickel bread, brown rice, other grains, bran, wheat germ, and popcorn |
| **Dairy** | **Low fat dairy** | Skim milk, sweetened or artificially sweetened yogurt, low-fat cheese (cottage/ricotta, other cheese), frozen yogurt, sorbet, low fat ice cream |
|  | **High fat dairy** | Whole milk, ice cream, high fat cheese (cottage/ricotta, other cheese), cream cheese, cream |
| **Seafood** | **Seafood** | Dark-meat fish (e.g., tuna steak, mackerel, salmon, sardines, bluefish, swordfish), canned tuna,  other fish (e.g., cod, haddock, halibut), shellfish (shrimp, lobster, scallops) |
| **Meat** | **Processed red meat** | bacon, salami, bologna, processed meat sandwiches, and other processed meats (e.g., sausage, kielbasa) |
|  | **Unprocessed red meat** | Beef as main dish, pork as main dish, beef, pork or lamb as a sandwich, hamburger (lean or regular) plus processed meat |
|  | **Chicken without skin** | Chicken or turkey hot dogs or sausage, chicken/turkey sandwich, other chicken or turkey without skin |
| **Legumes, nuts** | **Legumes** | string beans, peas, beans or lentils, soy, tofu |
|  | **Nuts** | peanuts, walnuts, other nuts |
| **Oils** | **Olive oils** | olive oils |
| **Beverages** | **Sugar-sweetened beverages** | Carbonated beverages with caffeine & sugar (e.g., Coke, Pepsi, Mt. Dew, Dr. Pepper), carbonated beverages with sugar (e.g., 7-Up, root beer, ginger ale, caffeine-free Coke), and other sugared beverages (e.g., punch, lemonade, sports drinks, or sugared iced tea) |
|  | **Wines** | red wine, white wine |
| **Other** | **Commercial bakery goods, sweets, and pastries** | Muffins or biscuits, candy bars, candy without chocolate, cookies or brownie, donuts, cake, pie, sweet rolls. |
|  | **Spread fats** | Butter, margarine, cream |

**Supplemental Table 3**. Summary of covariates in the Nurses’ Health Study (1994-2014) and Health Professionals Follow-Up Study (1994-2014) and in the parametric g-formula.

| **Variable name** | **How the variable was assessed** | **Years assessed** | **As dependent: the model form when the covariate is to be predicted by other covariates** | **As independent: the functional form when the covariate serves as a predictor to predict other covariates and the outcome of interest** |
| --- | --- | --- | --- | --- |
| **Time-fixed** |  |  |  |  |
| *Baseline non-dietary variables* |  |  |  |  |
| Baseline age | Self-reported | 1994 | Not predicted | Restricted cubic spline transformation with 4 knots |
| Family history of myocardial infarction before age 60 |  | 1994 | Not predicted | 2 categories |
| Family history of diabetes status |  | 1994 | Not predicted | 2 categories |
| Physical activity prior to baseline |  | 1994 | Not predicted | 3 categories |
| Regular aspirin use at baseline |  | 1994 | Not predicted | 3 categories |
| Current aspirin use at baseline |  | 1994 | Not predicted | 2 categories |
| Baseline log BMI |  | 1994 | Not predicted | Linear term |
| *Pre-baseline dietary variables* | Assessed via FFQ |  |  |  |
| Fruit including juice |  | 1990 | Not predicted | Linear term and restricted cubic spline transformation with 3 knots |
| Seafood |  | 1990 | Not predicted |  |
| High-fat dairy |  | 1990 | Not predicted |  |
| Vegetables (no legumes) |  | 1990 | Not predicted |  |
| Nuts (peanuts, walnuts, other nuts, including peanut butter) |  | 1990 | Not predicted |  |
| Processed meat |  | 1990 | Not predicted |  |
| Chicken without skin |  | 1990 | Not predicted |  |
| Whole grains |  | 1990 | Not predicted |  |
| Legume (peas, beans, string bean) |  | 1990 | Not predicted |  |
| Sugar-sweetened beverages |  | 1990 | Not predicted |  |
| Sweets (cake, cracker, muffin, chocolate, candies, donut, brownie, pie) |  | 1990 | Not predicted |  |
| Olive oil |  | 1990 | Not predicted |  |
| Spread fat |  | 1990 | Not predicted |  |
| Wine |  | 1990 | Not predicted |  |
| Total calories |  | 1990 | Not predicted |  |
| **Time-varying** |  |  |  |  |
| *Non-dietary variables* |  |  |  |  |
| Cigarette (current smoker) |  | 1994, 1996, 1998, 2000, 2002, 2004, 2006, 2008, 2010, 2012 | Logistic and then log-linear | Function of most recent lag, cubic (linear, squared and cubed terms) |
| Current aspirin |  |  | Logistic | Function of most recent lag, no transformation (binary, 2 categories) |
| Physical activity | Assessed using a validated questionnaire^[1]^ | 1994, 1996, 1998, 2000, 2004, 2006, 2008, 2010, 2012 | Logistic and then log-linear | Function of most recent lag, restricted cubic spline transformation (4 knots) |
| Log BMI | Self-reported | 1994, 1996, 1998, 2000, 2002, 2004, 2006, 2008, 2010, 2012 | Log transformed, Linear | Function of most recent lag, restricted cubic spline transformation (4 knots) |
| Reported High Blood Pressure | Self-reported |  | Logistic to failure; once switching from 0 to 1, stay 1 | An indicator of diagnosis and time since report of diagnosis |
| Reported High Cholesterol | Self-reported |  |  |  |
| Diabetes | Self-reported, followed by medical record review^[2]^ |  |  |  |
| Cancer | Self-reported, followed by medical record review |  |  |  |
| Post-menopausal hormones (only for NHS) | Self-reported |  | Logistic | No transformation (binary, 2 categories) |
| *Dietary variables* | Assessed via FFQ |  |  |  |
| Log calories |  | 1994, 1998, 2002, 2006, 2008, 2012 | Log transformed, linear | Restricted cubic spline transformation (3 knots) |
| Fruit including juice |  |  |  |  |
| Whole grains |  |  |  |  |
| Chicken without skin |  |  | Logistic and then log-linear |  |
| Sugar-sweetened beverages |  |  |  |  |
| Spread fat |  |  |  |  |
| Wine |  |  |  |  |
| Seafood |  |  |  |  |
| Nuts (peanuts, walnuts, other nuts, including peanut butter) |  |  |  |  |
| Legume (peas, beans, string bean) |  |  |  |  |
| Sweets (cake, cracker, muffin, chocolate, candies, donut, brownie, pie) |  |  |  |  |
| High-fat dairy |  |  |  |  |
| Olive oil |  |  |  |  |
| Vegetables (no legumes) |  |  |  |  |
| Processed meat |  |  |  |  |
| Remove fat from meat |  |  | Logistic | Function of most recent lag, no transformation (binary, 2 categories) |

**Supplemental Table 4**. Percentages of participants in the pooled cohort of Nurses’ Health Study (1994 – 2014) and Health Professionals Follow-Up Study (1994 – 2014) meeting each food recommendation in the low-fat diet, the Mediterranean diet, and the food-based AHA-2020 dietary goals.

| **Year** | **1994** | **1996** | **1998** | **2002** | **2004** | **2006** | **2008** | **2010** | **2012** | **2014** |
| --- | --- | --- | --- | --- | --- | --- | --- | --- | --- | --- |
| **The low-fat diet** |  |  |  |  |  |  |  |  |  |  |
| Fresh fruits ≥ 3 servings/day | 32.2 | 32.1 | 31.8 | 31.8 | 26.6 | 25.9 | 24.4 | 23.9 | 23.7 | 23.3 |
| Vegetables ≥ 2 servings/day | 77.3 | 77.5 | 71.4 | 71.6 | 65.7 | 65.5 | 61.6 | 61.6 | 58.6 | 59.3 |
| Exclusively low-fat dairy (%) | 75.8 | 75.8 | 70.2 | 70.1 | 59.2 | 58.5 | 58.8 | 59.0 | 56.6 | 56.4 |
| Seafood ≤ 1 serving/week | 32.3 | 32.3 | 43.2 | 43.7 | 47.5 | 47.7 | 50.3 | 50.0 | 51.4 | 51.1 |
| Remove fat from meat (%) | 98.5 | 98.5 | 98.3 | 98.4 | 98.1 | 98.1 | 97.7 | 97.6 | 97.6 | 97.8 |
| Processed meat ≤ 1 serving/week | 57.9 | 58.1 | 58.0 | 58.1 | 57.2 | 57.4 | 56.9 | 56.9 | 56.2 | 56.5 |
| Nuts ≤ 1 serving/week | 59.1 | 59.2 | 57.5 | 57.4 | 49.3 | 48.4 | 40.6 | 39.3 | 37.9 | 36.7 |
| Olive oils ≤ 1 tbsp/day | 96.1 | 96.0 | 94.8 | 94.7 | 93.2 | 92.9 | 93.2 | 93.1 | 93.1 | 92.9 |
| Commercial bakery goods, sweets, and pastries ≤ 1 serving/week | 24.7 | 24.8 | 27.8 | 27.8 | 29.7 | 29.8 | 30.7 | 30.6 | 34.8 | 35.3 |
| Spread fats ≤ 1 serving/week | 21.0 | 20.9 | 21.3 | 21.2 | 23.0 | 23.0 | 23.9 | 23.8 | 23.0 | 22.7 |
| *Meeting all above criteria simultaneously* | 1.4 | 1.4 | 2.0 | 2.1 | 2.3 | 2.3 | 2.0 | 1.9 | 2.1 | 2.0 |
| **The Mediterranean Diet** |  |  |  |  |  |  |  |  |  |  |
| Fresh fruits ≥ 3 servings/day | 32.2 | 32.1 | 31.8 | 31.8 | 26.6 | 25.9 | 24.4 | 23.9 | 23.7 | 23.3 |
| Vegetables ≥ 2 servings/day | 77.3 | 77.5 | 71.4 | 71.6 | 65.7 | 65.5 | 61.6 | 61.6 | 58.6 | 59.3 |
| Seafood ≥ 3 servings/week | 22.3 | 22.2 | 15.4 | 15.3 | 15.0 | 14.9 | 13.1 | 12.7 | 12.7 | 12.9 |
| Processed meat < 1 serving/day | 96.9 | 97.0 | 96.7 | 96.9 | 96.7 | 96.9 | 96.8 | 97.1 | 96.6 | 96.4 |
| Nuts ≥ 3 servings/week | 15.2 | 15.1 | 16.8 | 16.7 | 23.8 | 24.5 | 32.7 | 33.7 | 33.4 | 34.2 |
| Legume ≥ 3 servings/week | 34.9 | 34.9 | 29.6 | 29.4 | 28.4 | 28.2 | 28.1 | 27.7 | 28.4 | 28.4 |
| Olive oils ≥1 tbsp/day | 5.4 | 5.4 | 5.7 | 5.9 | 7.5 | 7.7 | 7.4 | 7.6 | 7.3 | 7.5 |
| Soft drinks < 1 drink/day | 93.4 | 93.4 | 92.2 | 92.1 | 92.2 | 92.3 | 94.9 | 95.1 | 95.0 | 95.0 |
| 1 ≤ Wine ≤ 3 drink/day (for baseline drinkers) | 59.2 | 59.2 | 59.3 | 59.1 | 58.7 | 58.5 | 58.0 | 57.4 | 56.8 | 55.7 |
| Commercial bakery goods, sweets, and pastries < 3 servings/week | 48.7 | 48.8 | 49.7 | 49.6 | 49.7 | 49.9 | 51.2 | 51.2 | 56.1 | 57.3 |
| Spread fats < 1 serving/day | 57.1 | 57.0 | 59.6 | 59.6 | 61.4 | 61.7 | 63.2 | 63.3 | 61.1 | 60.7 |
| *Meeting all above criteria simultaneously* | 0.01 | 0.01 | 0.01 | 0.01 | 0.03 | 0.04 | 0.05 | 0.06 | 0.07 | 0.06 |
| **The food based AHA 2020 dietary goals** |  |  |  |  |  |  |  |  |  |  |
| Fruits and vegetables ≥ 4.5 serving/week | 68.0 | 68.1 | 63.7 | 63.7 | 56.7 | 56.1 | 51.9 | 51.9 | 49.3 | 49.8 |
| Whole grains ≥ 3 servings/week | 86.6 | 86.7 | 85.0 | 85.2 | 86.2 | 86.4 | 86.8 | 87.4 | 88.8 | 89.7 |
| Seafood ≥ 2 servings/week | 36.4 | 36.3 | 27.6 | 27.2 | 25.2 | 25.0 | 23.2 | 22.9 | 22.3 | 22.6 |
| Processed meat ≤ 2 servings/week | 77.6 | 77.8 | 76.9 | 77.1 | 75.6 | 75.8 | 75.2 | 75.2 | 74.7 | 74.5 |
| Legumes and nuts ≥ 4 servings/week | 23.7 | 23.6 | 19.3 | 19.1 | 18.2 | 18.0 | 18.7 | 18.5 | 18.9 | 18.8 |
| Soft drinks ≤ 3 drinks/day | 81.2 | 81.2 | 78.3 | 78.1 | 78.6 | 78.7 | 83.9 | 84.6 | 85.2 | 85.4 |
| *Meeting all above criteria simultaneously* | 6.1 | 6.1 | 4.0 | 4.0 | 3.7 | 3.6 | 3.7 | 3.7 | 3.6 | 3.7 |

**Supplementary Table 5.** Sensitivity analysis: estimated risk of cardiovascular events under hypothetical dietary intervention strategies in the stacked ***high-risk*** population using pooled data from the Nurses’ Health Study (1994-2014) and Health Professionals Follow-Up Study (1994-2014), United States.

|  | **20-year risk of CVD (%) (95%CI)** | | | **Risk ratio (95% CI)** | | |
| --- | --- | --- | --- | --- | --- | --- |
|  | **The Low-Fat diet** | **The Mediterranean diet** | **The AHA-2020 dietary goals** | **The Mediterranean diet** | | **The AHA-2020 dietary goals** |
| (0) Primary Analysis | 35.9 (33.1, 38.5) | 28.2 (25.8, 31.4) | 31.1 (29.1, 33.4) | | 0.79 (0.70, 0.91) | 0.87 (0.79, 0.96) |
| (1) Further adjustment for screening^[3]^ | 35.6 (32.8, 38.4) | 28.4 (25.9, 31.8) | 31.1 (28.9, 33.5) | | 0.80 (0.71, 0.92) | 0.87 (0.79, 0.96) |
| (2) Adjustment for covariates from previous questionnaires rather than those concurrently measured with diet in a given questionnaire | 36.3 (33.6, 38.9) | 28.6 (25.9, 31.9) | 31.5 (29.3, 33.9) | | 0.79 (0.70, 0.90) | 0.87 (0.79, 0.95) |
| (3) Use of different functional forms for covariates | 35.6 (32.9, 38.7) | 27.7 (24.2, 31.8) | 31.1 (28.8, 33.4) | | 0.78 (0.67, 0.92) | 0.87 (0.79, 0.96) |
| (4) Change the order of covariates | 35.8 (33.1, 38.6) | 28.3 (25.7, 31.5) | 31.1 (29.1, 33.4) | | 0.79 (0.70, 0.91) | 0.87 (0.79, 0.96) |
| (5) Additionally include interaction terms between time-varying dietary variables | 36.0 (33.2, 38.5) | 27.8 (25.3, 31.2) | 31.0 (28.7, 33.3) | | 0.77 (0.69, 0.89) | 0.86 (0.78, 0.95) |
| (6) Redefine the primary CVD outcome as a composite of non-fatal MI, fatal CHD, and total stroke | 21.1 (18.8, 23.2) | 17.0 (14.7, 19.8) | 19.5 (17.9, 21.6) | | 0.80 (0.68, 0.97) | 0.92 (0.81, 1.07) |
| (7) Negative control outcome: basal cell carcinoma | 21.51 (19.72, 23.92) | 20.04 (17.43, 23.14) | 20.85 (19.06, 23.26) | | 0.93 (0.79, 1.09) | 0.97 (0.86, 1.10) |
| (8) Negative control outcome: squamous cell carcinoma | 19.49 (17.01, 21.96) | 21.55 (18.83, 24.95) | 24.52 (22.14, 26.89) | | 1.11 (0.96, 1.32) | 1.26 (1.12, 1.43) |
| (9) Negative control outcome: melanoma | 1.98 (1.41, 4.27) | 3.08 (1.96, 6.21) | 2.42 (1.76, 5.24) | | 1.55 (0.86, 2.68) | 1.22 (0.78, 2.04) |
| (10) Additional adjustment for beer and liquor | 35.3 (32.6, 38.0) | 28.5 (25.9, 31.8) | 30.9 (29.0, 33.3) | | 0.81 (0.72, 0.93) | 0.88 (0.80,0.97) |

**Supplementary Figure 1.** Directed acyclic graph.

Let t=0,1,…,9 denote each 2-year follow-up visit, where t=0 corresponds to 1994, t=1 to 1996, and so on. Let A_t_ denote the vector of dietary exposures at time t. Let V denote baseline covariates measured at or before t=0, including demographic characteristics, lifestyle factors, clinical history, and pre-baseline dietary intake (e.g., age, sex, BMI, physical activity, aspirin use, family history of disease, and prior diet).

Let L_t_ denote time-varying covariates at time t, including updated lifestyle factors and incident health conditions (e.g., smoking, physical activity, BMI, and diagnoses of hypertension, hypercholesterolemia, diabetes, and cancer). Let Y_t+1_ denote the indicator of the event of interest (CVD) by the end of interval t+1, and C_t+1_ the indicator of censoring due to loss to follow-up by the end of interval t+1. Let U denote unmeasured covariates that may affect time-varying covariates and outcomes.

**Supplementary Figure 2.** Flowchart of the high-risk the emulation of a target trial of dietary interventions in the Nurses’ Health Study (1994 – 2014) and Health Professionals Follow-Up Study (1994 – 2014), United States.

* Other medication conditions include Parkinson’s disease, amyotrophic lateral sclerosis, multiple sclerosis, Crohn’s disease, ulcerative colitis.

**Supplementary Figure 3**. Estimated cumulative risk of primary endpoint under dietary strategies consistent with the low-fat diet, the Mediterranean diet, and the food-based AHA 2020 dietary goals, in the pooled high-risk population using pooled data from the Nurses’ Health Study (1994–2014) and Health Professionals Follow-Up Study (1994–2014), stratified by baseline age, body mass index, and physical activity.

P-values for interaction were calculated using Wald tests; all interactions were non-significant, except for the comparison of AHA dietary goals versus low-fat diet by age.

**Supplemental Figure 4.** Flowchart of the general population (unselected on cardiovascular risk factors) for the emulation of a target trial of dietary interventions in the Nurses’ Health Study (1994 – 2014) and Health Professionals Follow-Up Study (1994 – 2014), United States.

* Other medication conditions include Parkinson’s disease, amyotrophic lateral sclerosis, multiple sclerosis, Crohn’s disease, ulcerative colitis.

**Supplementary Figure 5**. Evaluation of model misspecification in G-formula: Estimated natural course risk of the primary CVD outcome, risk of competing death, and mean level of covariates in the high-risk population in the pooled data from the Nurses’ Health Study (1994–2014) and Health Professionals Follow-Up Study (1994–2014), United States.

Solid line: estimates by inverse probability of weighting method; Dotted line: estimates by parametric G-formula method.

Right column: differences between IP-weighted and parametric g-formula natural course estimates and 95% confidence intervals.

**Abbreviation.**

act: physical activity

**Abbreviation.**

ca: confirmed cancer

chl: high cholesterol

**Abbreviation.**

cig: smoking

dia: diabetes

**Abbreviation.**

fruitj: fruit including juice

hbp: high blood pressure

**Abbreviation.**

hfdairy: high-fat dairy

lbmi: log BMI

**Abbreviation.**

lcal: log calories

lchicken: chicken without skin

**Abbreviation.**

legumesoy: legume (peas, beans, string bean)

nomeatfat: remove fat from meat

**Abbreviation.**

pmeat: processed red meat

**Abbreviation.**

sugbev: sugar beverage

sweet: bakery goods, sweets, and pastries

**Abbreviation.**

tnut: nuts (peanuts, walnuts, other nuts, including peanut butter)

vegNB: vegetables (no legumes)

**Abbreviation.**

wgrain: whole grains

wineu: wine, including red wine and white wine

1. Al-Shaar L, Pernar CH, Chomistek AK, et al. Reproducibility, Validity, and Relative Validity of Self-Report Methods for Assessing Physical Activity in Epidemiologic Studies: Findings From the Women's Lifestyle Validation Study. *Am J Epidemiol*. 2022;191(4):696-710. doi:10.1093/aje/kwab294
2. *Type 2 Diabetes Project in the Nurses’ Health Study and Health Professionals Follow-up Study: Study Protocol and Data Collection Instruments*. dbGaP. National Center for Biotechnology Information; July 2009. Document Accession phd000220.2; Study Accession phs000091.v2.p1. Accessed April 12, 2026. <https://www.ncbi.nlm.nih.gov/projects/gap/cgi-bin/document.cgi?study_id=phs000091.v2.p1&phd=220>
3. At least four of the following screening/examinations: routine physical examinations, rectal examination, eye examination, fasting blood sugar, mammography or sigmoidoscopy/coloscopy for screening
